# Microbiome Gut-Brain-Axis: Impact on Brain Development and Mental Health

**DOI:** 10.1007/s12035-025-04846-0

**Published:** 2025-04-15

**Authors:** Yasmin N. Ramadan, Saleh F. Alqifari, Khaled Alshehri, Amirah Alhowiti, Hyder Mirghani, Tariq Alrasheed, Faisal Aljohani, Abdulaziz Alghamdi, Helal F. Hetta

**Affiliations:** 1 Department of Microbiology and Immunology, Faculty of Pharmacy, Assiut University, Assiut, 71515 Egypt; 2 Department of Pharmacy Practice, Faculty of Pharmacy, University of Tabuk, 71491 Tabuk, Saudi Arabia; 3 Department of Internal Medicine (Neurology), Faculty of Medicine, University of Tabuk, Tabuk, Saudi Arabia; 4 Department of Family and Community Medicine, Faculty of Medicine, University of Tabuk, Tabuk, Saudi Arabia; 5 Department of Internal Medicine, Faculty of Medicine, University of Tabuk, Tabuk, Saudi Arabia; 6 Division of Medicine and Gastroenterology, Department of Medicine, Faculty of Medicine, University of Tabuk, Tabuk, Saudi Arabia; 7 Department of Medicine, Division of Psychiatry, Faculty of Medicine in Rabigh, King Abdulaziz University, Jeddah, Saudi Arabia; 8 Division of Microbiology, Immunology and Biotechnology, Department of Natural Products and Alternative Medicine, Faculty of Pharmacy, University of Tabuk, 71491 Tabuk, Saudi Arabia

**Keywords:** Gut microbiome, Gut-brain axis, Brain development

## Abstract

The current discovery that the gut microbiome, which contains roughly 100 trillion microbes, affects health and disease has catalyzed a boom in multidisciplinary research efforts focused on understanding this relationship. Also, it is commonly demonstrated that the gut and the CNS are closely related in a bidirectional pathway. A balanced gut microbiome is essential for regular brain activities and emotional responses. On the other hand, the CNS regulates the majority of GI physiology. Any disruption in this bidirectional pathway led to a progression of health problems in both directions, neurological and gastrointestinal diseases. In this review, we hope to shed light on the complicated connections of the microbiome-gut-brain axis and the critical roles of gut microbiome in the early development of the brain in order to get a deeper knowledge of microbiome-mediated pathological conditions and management options through rebalancing of gut microbiome.

## Introduction

“All disease begins in the gut”; this statement is said to have been stated by the Greek doctor Hippocrates, who is sometimes named with father of modern medicine, more than 2000 years ago. Whether or not Hippocrates is the true author is debatable, yet its innate wisdom still has an impact on medical researchers and physicians nowadays [1].

The recognition that the brain and gut interact in continuous, bidirectional interaction dates back to Ancient Greece, when scientists such as Hippocrates, Plato, and Aristotle proposed that the brain and other parts of the body are fundamentally linked. This idea led to the realization that studying the processes of illness requires considering the full person instead of just one or two separate organ systems [2]. Nonetheless, William Beaumont conducted experiments before the 1840s that demonstrated how emotional state impacted the pace of digestion, indicating that the brain influences the gut and that a brain-gut axis exists. Even though this notion was thereafter recognized by Darwin, Pavlov, James, Bernard, and Cannon [3], it took until the beginning to mid-twentieth century for the initial scientifically documented findings that associated gut physiology modifies alongside swings in emotion. However, due to primitive technologies and a scarcity of research on the reciprocal consequences of altered gut physiology on brain performance, these investigations were constrained. Recent research has supported the links between gut and brain health [4]. Much research found a high correlation between a variety of host illnesses and alterations in the microbiome composition, or “dysbiosis” [5, 6]. It has been reported that any alteration in the gut microbiome may be linked to developing a variety of CNS illnesses. Furthermore, the reciprocal connections can now be seen for the first time thanks to the advancement in brain imaging, revealing that important brain areas that participate in emotion regulation may be activated by gut signals [3].

The human gut microbiome is a rich and diverse ecosystem made up of commensal bacteria, viruses, fungi, and archaea [7–10]. This ecosystem starts to colonize the GI during in-utero life [11]. The growth and colonization of gut microorganisms co-occur alongside brain growth during pregnancy and continue up to a few years after delivery. Throughout the initial 12 months of postnatal development, the microbiome composition differs greatly between individuals until it stabilizes and resembles an adult at around 3 years of age [12–15]. This variability may affect the development of the brain and shape an individual’s immune profile. In a healthy individual, early mucosal colonization is critical for the formation and maturation of the host’s immune system [13]. Early childhood gut dysbiosis, however, can result from exposure to conditions including maternal immune activation (MIA), improper nutrition, illness/infections, and antibiotic overuse [16, 17]. The disrupted gut microbiome can promote inappropriate immune function, leading to systemic inflammation and symptoms linked with neurodevelopmental psychiatric disorders (NPD) [18–20]. Therefore, the effectiveness of the immune system, which subsequently controls neurodevelopmental pathways, depends on the existence of a balanced microbiome [21, 22].

Despite rising data, there is still a considerable gap in knowing the precise mechanisms that regulate the connection between GIT and the brain through health and illness. This review will shed a spotlight on the complex links of the microbiome-gut-brain axis and the critical roles of gut microbiome in early brain development to gain a deeper understanding of microbiome-mediated pathological conditions, noninvasive prognostic pathways, and management options utilizing microbiome-gut-brain-axis adjustments.

## Gut-Brain Axis

The gut forms a complex, bidirectional link with the CNS, known as gut-brain axis, active in both health and illness [3]. This interaction enables gut sensory impulses, transmitted via the vagus nerve, to impact CNS activity, controlling reflexes and modulating mood. The brain then uses these signals to alter gut physiology as well as other functions. Signals are transmitted through pathways like the enteric nervous system (ENS), autonomic nervous system (ANS), hypothalamic-pituitary-adrenal (HPA) axis, sympatho-adrenal axis, and descending monoaminergic pathways, involving both afferent (signal-receiving) and efferent (signal-sending) neurons [3, 23]. Multiple inter-relational and neurohumoral elements regulate and closely connect to each pathway. In significant part, the innate innervation of gut functions is mediated by the intricate neuronal network known as the ENS. It is composed of the myenteric and submucosal plexuses, two ganglionated plexuses that control gut peristalsis, absorption, and secretion [24]. In gut-brain communication, the ENS sends signals to the CNS through intestinofugal neurons that connect to the sympathetic nervous system (SNS), while sensory information travels via vagal afferent pathways [24].

The ANS is a network of sympathetic and parasympathetic neurons [3]. ANS regulates respiration, heart rate, and CNS-mediated alterations in the GIT and related processes, including digestion, GI motility, and permeability, bile secretion, carbohydrate metabolism, mechanical mucosal distortion, luminal osmolality, preservation of epithelial fluid balance, mucus production, as well as mucosal immune response [3, 25]. The CNS sends direct signals from the ANS to the gut, affecting its physiology. The gut microbiome communicates through metabolites that are recognized by host cells, which then interact with ANS synapses in the gut [26]. Additionally, the ANS can influence the gut epithelium, impacting immune system activation either by directly altering immune cell responses to the microbiome or by changing how the microbiome interacts with immune cells [27, 28].

## Microbiome Gut-Brain Axis (MGBA)

**Fig. 1:**
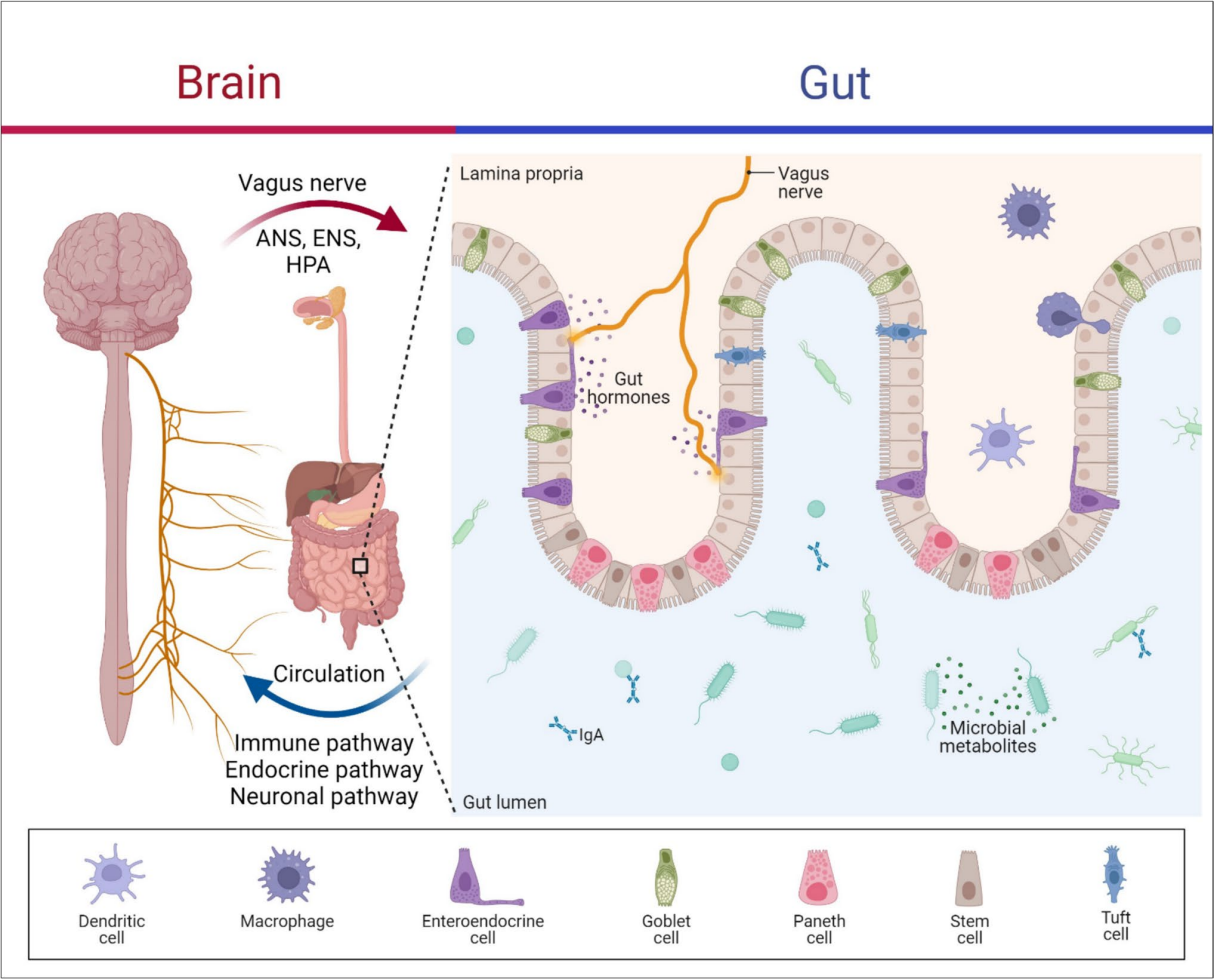
Microbiome gut-brain axis. The brain can affect the gut through various pathways, like the enteric nervous system (ENS), autonomic nervous system (ANS), hypothalamic–pituitary–adrenal (HPA) axis, sympatho-adrenal axis, and descending monoaminergic pathways, involving both afferent (signal-receiving) and efferent (signal-sending) neurons. On the other hand, the gut impacts CNS function through microbiome metabolites, neuroactive agents, and gut hormones that reach the brain through the vagus nerve, circulatory system, immune system, or ENS. Created with BioRender

The signaling pathway underlying the connection between the gut-brain axis and the microbiome is very important when thinking about therapeutic approaches. The brain influences gut functions via the HPA axis and the ANS; for instance, norepinephrine (NEP) is produced by the brain under stress and has been shown to promote the proliferation of gut pathogens [29]. On the other hand, the gut impacts CNS function through microbiome metabolites, neuroactive agents, and gut hormones that reach the brain through the vagus nerve, circulatory system, immune system, or ENS (Figure 1) [30, 31].

Together, gut hormones, neuroactive compounds, and microbial metabolites form a complex signaling network that directly impacts brain function and mental health. For instance, short-chain fatty acids (SCFAs) like butyrate and propionate, produced through microbial fermentation, not only maintain gut integrity but also influence brain functions such as mood regulation and cognitive processes. SCFAs cross the blood-brain barrier (BBB) and interact with receptors like GPR41 and GPR43, modulating neuroinflammation and neurotransmitter release [32].

Similarly, gut-derived serotonin and dopamine play dual roles. Enterochromaffin cells produce serotonin in the gut, which affects not only gut motility but also transmits signals to the CNS via the vagus nerve. Deficiencies in gut-derived serotonin are linked to mood disorders such as depression. Neuroactive compounds like gamma-aminobutyric acid (GABA), synthesized by gut bacteria, impact stress response and anxiety through the vagal pathway [33]. By integrating these pathways, we can better understand the gut microbiome’s central role in mental health disorders.

These pathways are collectively known as the MGBA. Interestingly, the vagus nerve is the most direct link out of all the potential paths since various receptors on the vagal afferents sense and send signals from the gut to the brain. Actually, it has been discovered that the vagus nerve affects CNS reward neurons, which in turn affects CNS mood and behavior [34]. The microbiome is essential for the induction, regulation, and function of both systemic and local immune responses, including innate and adaptive immunity. This makes the gut-immune-brain connection critical to overall health. Pre-clinical models have confirmed this, as germ-free (GF) mice exhibit significant immune deficiencies, such as enhanced vulnerability to infections due to decreased Peyer’s patch size and function, absence of the mucous layer, changed IgA secretions, and decreased proinflammatory T helper (Th) cell secretions [35, 36].

### Gut Microbiome-Derived Metabolites

Significant parts of the MGBA are the metabolites and products derived from the microbiome and mostly work via receptor-mediated interactions on a variety of host cells or tissues. SCFAs and endogenous tryptophan are two of the most well-studied metabolites. SCFAs—a byproduct of the microbial breakdown of carbohydrates—have been proposed to support glucose homeostasis, lymphocyte function, mucosal serotonin secretion, and learning and memory acquisition through maintaining BBB integrity [36]. Studies are few, but it is expected that SCFAs can potentially penetrate the BBB because the metabolite can be identified in human CSF and SCFA absorption was successfully seen in rats following the injection of labeled, ^14^C, SCFAs into the carotid artery [37]. GF animals showed increased BBB permeability, which supports the significance of SCFAs in CNS homeostasis. In contrast, BBB integrity is restored when the mice are recolonized with SCFA-producing bacteria [32]. Mechanistically, research has shown that SCFAs interact with G-protein coupled receptors (GPR) for a range of various functions, including GPR41 in enteric neurons and GPR43 in adipose tissues [38]. However, the findings have been inconsistent. For instance, acetate, a prominent SCFA from the gut microbiome, has been shown to control food intake in one study [39] yet inferred to boost food intake via ghrelin secretion in another [40].

It is also important to discuss how dietary tryptophan is converted by microbes into indole compounds. Recently, it was discovered that some bacteria, specifically those belonging to the *Lactobacillus* genus, are essential for activating the aryl hydrocarbon receptor, which in turn regulates the cell cycle and promotes T-cell differentiation [41]. Significantly, research has shown how dietary tryptophan is crucial for encephalitogenic T cell responses, which trigger CNS autoimmunity [42].

### Gut Microbiome-Derived products

Additionally, microbiome-derived products are important in MGBA communication. They do this frequently by interacting with toll-like receptors (TLR) in the CNS and ENS, which can detect the products by their molecular patterns. For instance, it has been demonstrated that (TLR-4), which is mostly found on CNS microglia, may detect lipopolysaccharide (LPS), the substance secreted by Gram-negative bacteria. This recognition in turn stimulates the synthesis and proliferation of proinflammatory cytokines [43, 44]. Significantly, this immune response has caused neuroinflammation, activation of microglia, and death of neural cells, all of which have contributed to cognitive disorders and have been linked to anxiety and depression [45]. Polysaccharide A, a different important microbiome-derived product, is produced by *B. fragilis* and identified by TLR-2, inducing a protective CNS anti-inflammatory effect [46].

In summary, the interactions between microbiome-derived metabolites and products within the MGBA are incredibly complex and varied. For example, a single metabolite may interact with several receptors located in various tissues and cell types, leading to a variety of physiological, immunological, and CNS reactions. In the future, medical treatment with SCFAs may be a specifically viable choice, and investigations in models of Parkinson’s disease [47], Alzheimer’s disease [48], multiple sclerosis [49, 50], and autism spectrum disorder [51] are currently ongoing, but with mixed results.

### Gut Hormones

Gut hormones also play a crucial role to take into account in gut-brain transmission. There are a number of gut hormones (such as ghrelin, CCK, and 5-HT) that are correlated with anxiety and depression, which supports the supposed associations between obesity and mood disorders [52]. One of the best-studied gut hormones, 5-HT has a broad range of receptor subtypes and locations. It has been shown that 5-HT when synthesized by enterochromaffin cells (EECs) stimulates the release of cytokines from lymphocytes and monocytes and can communicate with the CNS by stimulating vagal sensory afferents [53, 54]. Therefore, it is important to note that the microbiome plays a major role in the synthesis and release of gut hormones. For instance, GF mice have much lower 5-HT and dopamine levels than normal, and it was discovered that GLP-1 secretion is facilitated by indirect interactions with LPS and SCFAs, respectively [55, 56]. But it is also crucial to remember that gut hormones can have an impact on the microbiome. For example, 5-HT can be produced and secreted into the gut lumen by EECs, which changes the gut microbiome profile [56]. Thus, to inform the development of CNS therapeutics, it is critical to distinguish between the causes and effects of the interaction between gut hormones and gut microbiome going ahead and to interpret the research findings.

### Neuroactive Compounds

**Fig. 2:**
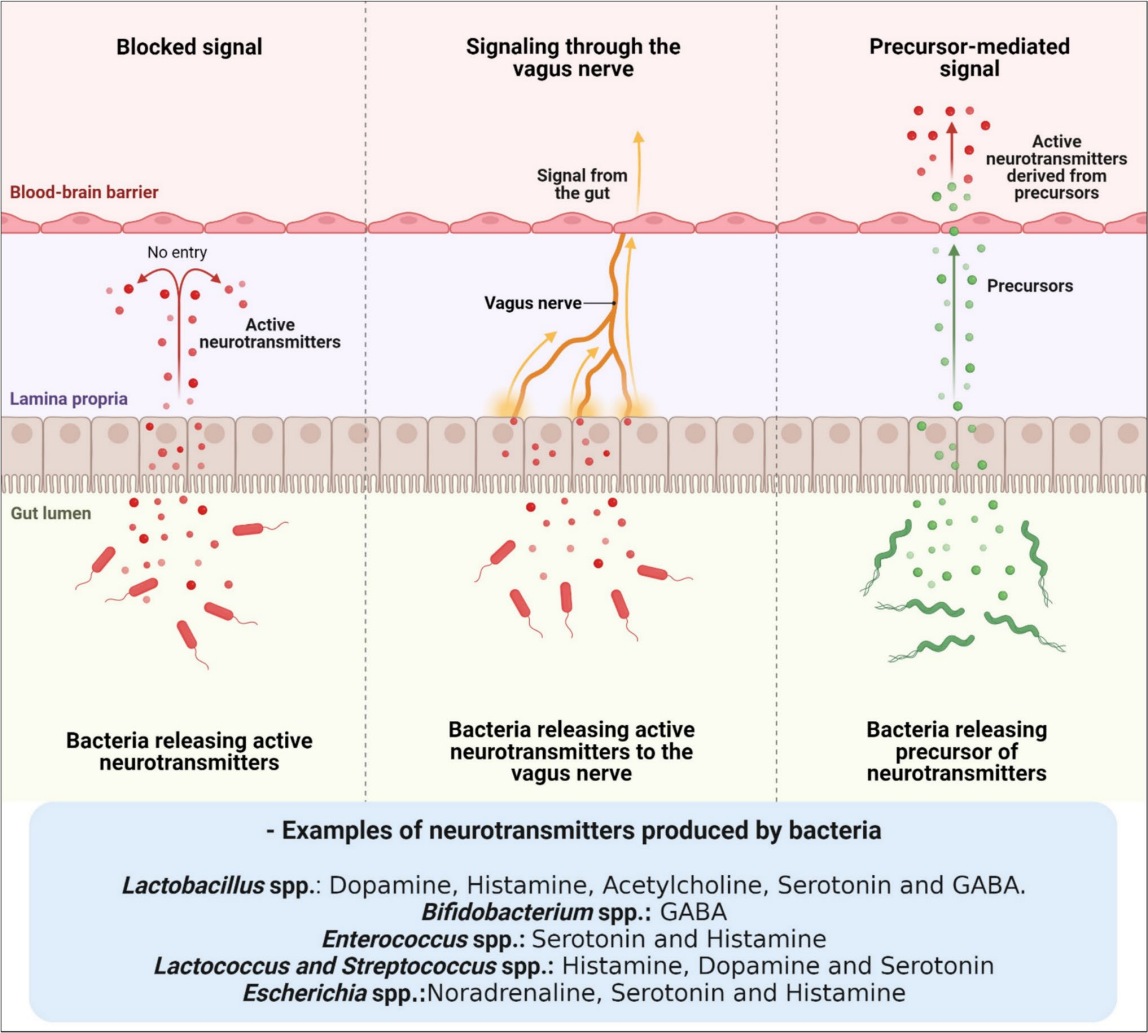
Different pathways of gut microbiome-derived neurotransmitters. Several neurotransmitters have been identified from different microbiome spp. found in the human gut. The synthesis of neurotransmitters by microbes offers a possible route of direct brain and behavioral modulation. Actually, this pathway is restricted as the majority of neurotransmitters—such as GABA, dopamine, and serotonin—generally cannot cross the BBB. Other potential pathways include the idea that neurotransmitters produced from microbiomes influence the brain via the vagus nerve and its afferent neurons. An alternative pathway is that neurotransmitter precursors are transformed into active neurotransmitters after passing through the BBB. For instance, tryptophan’s availability and metabolism—a precursor to serotonin—can be influenced by gut microbiome. Given that there is a correlation between brain serotonin levels and tryptophan content in the bloodstream, this might have an impact on serotonergic signaling in the CNS (modified from [ 33 ], created with BioRender)

Another category of gut microbiome-associated compounds that regulate the MGBA via the ENS is neuroactive compounds (Figure 2). The gut microbiome has been shown to regulate, and possibly produce, neuroactive compounds like acetylcholine, noradrenaline, dopamine, histamine, GABA, and melatonin, which in turn influence the CNS [5, 28]. Since these neuroactive compounds cannot cross the BBB, it is yet unclear how they impact the CNS [31]. More research into the mechanism of gut-modified neuroactive compounds is required.

### Direct Microbiome Invasion

Microorganisms may also directly invade the blood-brain barrier (BBB), but the exact pathways and entry points remain unclear. While the bacterial load and duration required for invasion vary by pathogen, a key factor in bacterial pathogenesis is often the colonization of mucosal surfaces and prolonged survival in the bloodstream. Here, bacteria can evade phagocytosis and other immune defenses before reaching brain endothelial cells and ultimately breaching the BBB through various pathways [57]. After bacterial adherence to the brain endothelium, bacteria might cross the endothelium by a paracellular route through breaking intercellular connections, a transcellular route through brain endothelial cells, or possibly a Trojan-Horse mechanism through infected phagocytes [58]. However, it is important to keep in mind that many results are still uncertain. For instance, the validity of in vitro studies on pathogen adhesion in the body is still debated, as bacteria need to survive blood flow in vivo. Nevertheless, BBB invasion continues to be a major clinical concern when treating neurological disorders. Therefore, understanding how bacteria survive through direct interactions with CNS barriers is key to developing better therapeutic strategies.

## Role of Gut Microbiome in the Early Development of the Brain

**Fig. 3:**
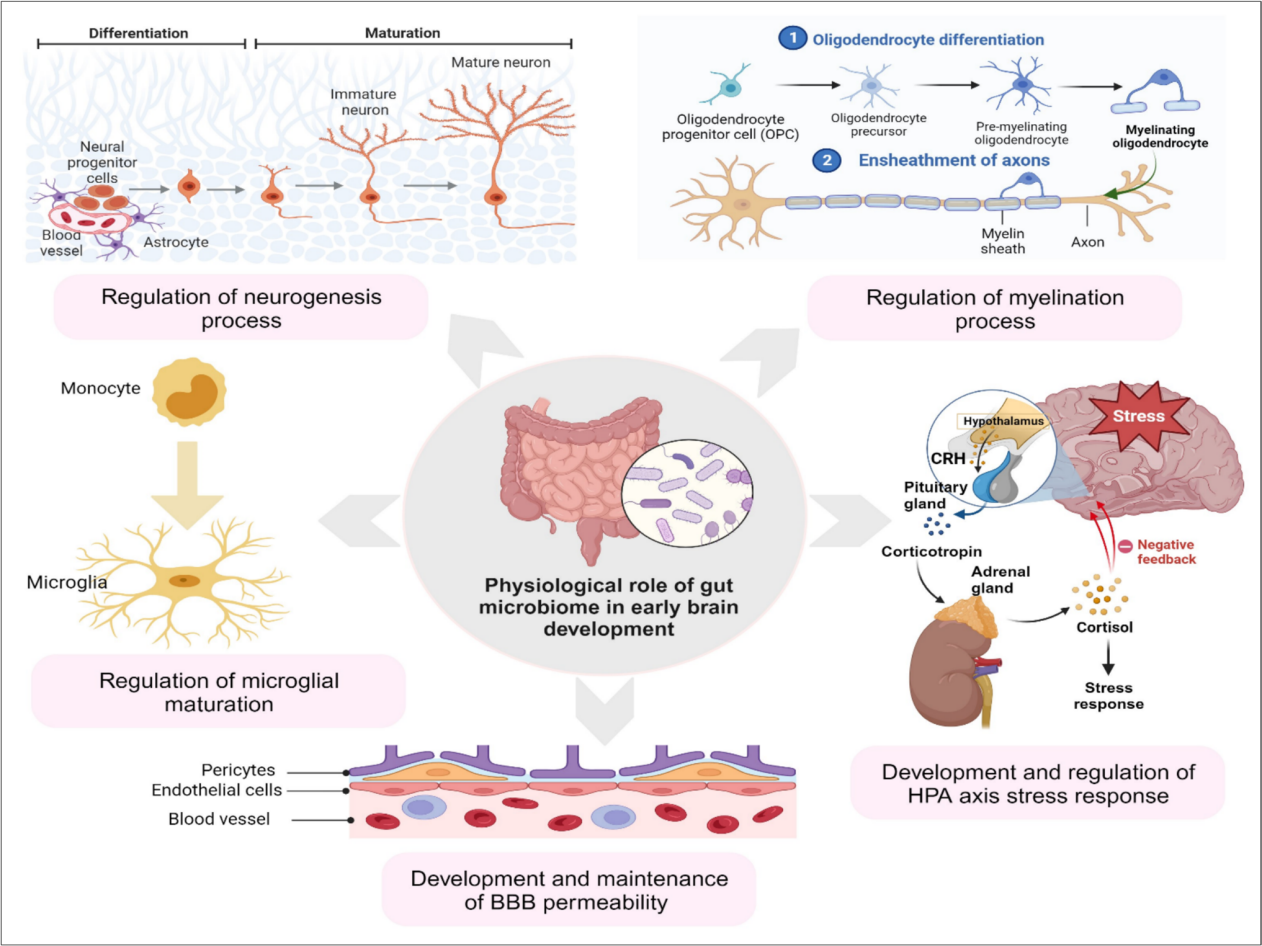
The role of the gut microbiome in early brain development. The gut microbiome is crucial for several stages of brain development, including regulation and maintenance of BBB permeability, neurogenesis, microglial maturation, myelination, HPA axis development, and HPA axis stress response. Any changes in this developmental phase can raise the risk of neurodevelopmental diseases considerably (modified from [115], created with BioRender)

The beginning of colonization and development of the gut microbiome occurs concomitantly with brain development during the pregnancy and continues until a few years after delivery [59, 60]. During the crucial embryonic stage, an imbalance in the gut microbiome might affect the entire developmental process, particularly the maturation and growth of neurons and glia [61]. Recent research indicates that the microbiome actively participates in the development of the CNS. Gut microbiomes are now known to actively participate in several neurodevelopmental processes, such as neurogenesis [62], myelination [63, 64], formation of the BBB [65], microglial maturation [66] as well as hypothalamus pituitary adrenal axis [67] (Figure 3). Our cognition and behavior are greatly influenced by these processes. The maturation and appropriate functioning of neuronal cells in the growing brain depend on a variety of dietary molecules and metabolites secreted from the gut [68, 69]. Furthermore, different research suggested that gut microorganisms might directly boost brain development processes, with long-term health consequences [18]. A person’s immune profile and processes related to brain development can be influenced by their microbiome composition, which varies most within and between individuals during the first 12 months after delivery and stabilizes at around 3 years of age [12, 13]. For a healthy individual, early colonization of the mucosal surfaces is crucial for the immune system’s development and maturation [13].

One the other hand, gut dysbiosis in infancy may be caused by several factors including excessive use of antibiotics, illness, infections, and maternal immune activation (MIA) [16, 17, 70]. The dysregulated immune activation resulting from altered gut microbiome can promote systemic inflammation and abnormal brain development, which can produce symptoms linked to neurodevelopmental psychiatric disorders (NPD) [18, 19, 21]. Accordingly, proper immune system functions, which in turn control neurodevelopmental pathways, depend on the existence of a balanced microbiome [13, 21, 22].

### Neurogenesis

The term “neurogenesis” describes the process by which neural stem/progenitor cells differentiate into new, functioning neurons [71]. This neurogenesis process is crucial for cognition, memory, learning, and stress response, particularly in the hippocampus, which serves as the cognitive center [72].

A balanced gut microbiome has a direct or indirect role in maintaining the milieu necessary for neural development [73]. Salvo et al. demonstrated that microbiome dysbiosis in pediatric animal models of inflammatory bowel disease (IBD) may result in behavioral deficits, decreased neurogenesis, neuroinflammation, and altered expression of pattern recognition receptor genes in the hippocampus [74]. In another animal study, a comparison of gut microbial metabolites in germ-free (GF) and specific pathogen-free (SPF) mice reveals a variety of substances that can stimulate and control the prenatal developmental process and penetrate the placenta into the fetal area [75].

Moreover, it has been reported that peptidoglycan can cross the placenta and enter the fetal brain, where it stimulates Toll-like receptor 2 (TLR2). This increases the expression of FOXG1, a transcription factor that is essential for controlling neurogenesis and development, ultimately leading to the proliferation of neurons in the forebrain region [76, 77]. Neuronal plasticity and maturation are linked to the process of synapse formation and maturation. It has been observed that the experimental delivery of neonatal prebiotics to 22-day-old rats, as opposed to other prebiotics, increases the levels of synaptophysin and brain-derived neurotrophic factor (BDNF) in the hippocampus [78].

Additionally, the gut microbiome may have an indirect impact on neuronal plasticity by controlling neuronal migration and maturation in the CNS. This could happen through the regulation of the reelin and ephrin B pathway, wherein reelin, a membrane glycoprotein responsible for neuronal migration, and ephrin B play a crucial role in maintaining the integrity of the gut epithelial barrier [79–82].

The regulation of adult neurogenesis by gut microbiome has also been documented in a number of investigations. In this regard, Ogbonnaya et al. showed that when proliferating cells in the brain of GF mice were labeled with bromo-deoxyuridine, an increase in adult dorsal hippocampus neurogenesis was seen as compared to conventionally raised animals, and the phenotypical state could not be reversed even after microbial colonization. This suggests that the lack of microorganisms causes an abnormal rise in adult dorsal hippocampal neurogenesis and that microbiological signals during a critical early developmental period influence hippocampal neurogenesis [83]. Additionally, gut microorganisms have the ability to produce and release serotonin into the gut lumen, which is known to stimulate adult neurogenesis [84].

### Myelination

A balanced gut microbiome has been documented to influence myelination. Usually, humans are born with unmyelinated axons in the CNS [63]. Within a few years after birth, oligodendrocytes quickly myelinate growing axons by engaging and enveloping them in a mechanism that varies in both myelination rate and myelin content until early adulthood [85–87]. Any deviation from this procedure might result in persistent problems. Cognitive function is primarily dependent on myelination, and neuronal plasticity and function have been related to the degree of myelination [88, 89]. The gut microbiome modulates the crucial process of myelination by influencing the expression of myelination-related genes in oligodendrocytes. Deformities of the myelin sheath can negatively affect behavior and brain function [63, 90]. Specifically, the brain’s prefrontal cortex (PFC) region myelinates later in infancy, during the early stages of development, leaving it more susceptible to outside influences such as intestinal dysbiosis. For instance, in GF mice, the myelin development in the PFC area is distorted which negatively impacts social behavior [63, 91]. In addition, bacterial metabolites such as SCFAs have been reported to be advantageous in regulating the myelination process, intestinal barrier malfunction and behavioral issues brought on by stress [91, 92]. Moreover, Oral administration of SCFA butyrate resulted in a restoration of intestinal physiology, behavioral deficit, and myelination impairments in mice treated with antibiotics. This suggests that the gut microbiome plays a crucial role in establishing the microbiome-gut-brain (MGB) axis by regulating the myelination process in the PFC region [93]. Therefore, the microbiome plays a critical role in both myelination and the preservation of the myelin sheath’s flexibility.

### Formation of Blood–Brain Barrier

The blood-brain barrier (BBB), the narrow barrier separating the brain from the systemic circulation, is formed early in pregnancy by capillary endothelial cells secured by tight junction proteins, astrocytes, and pericytes [32]. It also allows the interchange of elements and nutrients required for appropriate brain upkeep and function [94]. To maintain and control the development of an intact BBB, a balanced gut microbiome and its metabolites, such as SCFAs, are necessary [32, 94, 95]. BBB permeability declines with age in growing sterile babies [96]. It has been demonstrated that the permeability of the BBB for molecules rises in germ-free (GF) mice owing to lower levels of the crucial junctional proteins claudin-5 and occludin in the brain endothelial layer. Additionally, the permeability of BBB in GF mice was achieved by microbial colonization of the gut or by administering butyrate, SCFA generated by gut microbial fermentation [32].

### Microglial Maturation

The glial system contains resident immune cells called microglia (macrophages). They are widely dispersed throughout the brain and spinal cord [97] and make up about 10–15% of the total number of glial cells in the CNS [98]. In contrast to neuronal cells, microglial cells that comprise the CNS’s intrinsic immune system are produced from a subgroup of naive macrophages that start from the yolk sac progenitor cell [99–101]. Microglia are involved in both immunological defense and CNS maintenance. Microglial cells, for example, continuously monitor their local surroundings to identify pathogenic infiltration or tissue damage throughout the CNS, which is protected by the BBB [100, 102]. Inflammation is aberrantly induced by abnormal microglia activation, and this is seen in the majority of brain-related diseases. According to accumulating data, microglia have a direct impact on neuronal dysfunction and lead to disease development [103–106]. Recent studies have revealed the critical function of gut microbiome in the growth and maturation of microglia [66, 107–109]. Microglial cells in GF mice have a dramatically impaired developmental state, together with morphological features and a gene expression pattern associated with a developmental and maturation stall instance. These GF mice-derived microglia had a restricted response to infections which was significantly restored by SCFA treatment [110]. Moreover, bone marrow-derived macrophages can replace damaged or depleted microglial macrophages and yolk progenitor cells by boosting their growth and differentiation with the aid of early signals from the gut microbiome [66, 111].

### Hypothalamus Pituitary Adrenal (HPA) Axis

The HPA axis refers to the endocrine-neurocrine interaction between the hypothalamus, pituitary, and adrenal gland as a consequence of stress. The corticotropin-releasing factor (CRF) is one of the key players in this process because it triggers a series of events that cause the adrenal cortex to release glucocorticoids by controlling the HPA axis [112]. The development of the HPA axis is also significantly influenced by the gut microbiome [67]. For instance, GF mice’s hypothalamus showed higher levels of CRF mRNA in contrast to specific-pathogen-free (SPF) animals, suggesting an improved stress response connected to the HPA-axis [113]. Another study found that giving a probiotic mixture containing *Lactobacillus helveticus* and *Bifidobacterium longum* significantly reduced anxiety levels [114].

## Microbiome Gut-Brain Axis Throughout the Life Cycle

The gut microbiome has served as our lifelong friend throughout the journey of development. This diverse ecosystem is in continual fluctuation throughout our lifecycle [116]. Minor daily differences in the microbiome composition are typically seen among people, but these shifts become most apparent when we look throughout our lifetime. In terms of both diversity and prevalence of certain taxa, the microbiome at the two extremes of life differs noticeably from the usual adult gut microbiome [117–119].

### Early Life

The first colonization of the human GI tract is a topic of debate. Some recent studies have shown that there is a placental microbiome and that the fetus is colonizing the GI tract in-utero [11, 120, 121]; others, however, claim that the uterus and placenta are sterile [122–125]. If in-utero colonization occurs, it appears to possess a minimal impact on the composition of the early postnatal microbiome compared to the initial inoculation of the microbiome at birth.

It is challenging to define exactly what makes a healthy microbiome in the early stages of development. Nonetheless, it is well recognized that the microbiome typically develops along one of a few pathways, with early-colonizing species affecting the long-term makeup [126]. *Enterobacteriaceae*, *Bifidobacteriaceae*, and *Clostridiaceae* are usually found in relatively large levels in the microbiome shortly after birth, whereas *Lachnospiraceae* and *Ruminococcaceae* are found at lower levels [127–129]. As the baby grows, strict anaerobes eventually constitute dominant taxa. Around 1–3 years of age, when the baby starts to wean and consume solid food, the total diversity increases to adult-like levels [12, 130]. Generally, children’s gut microbiome is significantly enriched in pathways that promote continual growth (such as genes involved in de novo folate synthesis, vitamin synthesis, and anti-inflammatory pathways) [131].

The GF mice have been established to be very useful in understanding the function of the microbiome in gut-brain transmission throughout life [132]. In fact, in pregnant rodents, the GF status has a significant impact on the development of offspring. For instance, in the mouse, the BBB normally establishes throughout the second week of pregnancy, with permeability reducing rapidly around embryonic day 15. Significantly, this study found that postnatal recolonization of the microbiome might reestablish BBB integrity, suggesting a significant function of the microbiome in establishing BBB formation [32]. In conjunction with the mother’s GF status, additional prenatal maternal characteristics have also been discovered to affect the makeup of the child’s microbiome in rats and/or humans. These factors include nutrition [133], obesity [134], immunological activation [135], and stress [136, 137]. All of these factors are known to change both the physical and mental outcomes of the offspring [138].

In humans, the evidence suggesting long-term impacts of early life microbiome alterations on host physiology and brain health is mostly correlated. This evidence reveals a relationship between early-life microbiome composition and later metabolic and immune response [139–142]. There has been encouraging evidence from early clinical trials that probiotic therapies for children at risk can lower the likelihood of GIT issues [143–145], sepsis [146–148], and even attention deficit hyperactivity disorder (ADHD) [149] and autism spectrum disorder [149, 150]. Additionally, early life gut microbiome plays a critical role in shaping the brain’s neural circuits and stress response systems. Disruptions during this period can have lasting effects on cognitive function and mental health. Recent research [151] shows that early dysbiosis impacts neurogenesis, while Lynch et al. [152] emphasize its role in synaptic formation during critical developmental stages. Such studies are becoming increasingly common due to substantial preclinical evidence that early-life alteration of the microbiome affects a broad spectrum of behavioral and neurological consequences earlier in development and later in life [5, 153–160].

### Adolescence

Adolescence is a period of significant brain development, including synaptic pruning, myelination, and hormonal changes. These processes shape cognitive function, behavior, and stress responses. Given the rapid brain remodeling and changes in social and lifestyle habits during adolescence, it is not surprising that this period is associated with increased vulnerability to mental health issues like anxiety and depression. [161–164]. [165–167]. Changes in the gut microbiome during this critical phase are thought to influence these developmental processes. Studies show that disruptions to the MGBA in adolescence can alter social behavior and stress responses well into adulthood [152].

*Prevotella* and *Sutterella* were less common in teenagers, although *Bifidobacterium* and *Clostridium* were more common. Changes in microbiome composition during adolescence, including shifts in microbial diversity, are linked to developmental brain processes, but more research is needed to understand how these changes influence behavior and mental health.[168]. Research on mice has demonstrated that sex differences in the microbiome do not appear until puberty [169] and that probiotic administration during early life stress overcomes stress-induced alterations in the onset of puberty [170]. Moreover, adult cognition, social behavior, and anxiety are changed by prolonged antibiotic-induced dysbiosis of the microbiome in adolescence. Additionally, this long-term medication changed the way that adults metabolize tryptophan and decreased brain levels of oxytocin, vasopressin, and BDNF [158, 171, 172].

### Aging

Aging impacts the gut microbiome, contributing to cognitive decline and neuroinflammation. However, these age-related changes often stem from the early-life gut microbiome, which lays the foundation for long-term brain health. Early gut colonization influences neuroimmune regulation and stress response systems, which continue to affect brain resilience throughout life [151, 152].

Disruptions in early microbiome composition can lead to long-term neurodevelopmental consequences, increasing susceptibility to neurodegenerative conditions such as Alzheimer’s disease (AD) and Parkinson’s disease (PD) in older age [173–175]. For example, early dysbiosis may alter SCFA production, impairing neuroinflammation control and BBB integrity—both critical factors in age-related neurodegeneration [176].

Although aging is associated with a decline in beneficial bacteria like *Lactobacillus* and *Bifidobacterium*, these changes are often linked to the initial microbiome composition established in infancy and adolescence. Addressing gut health early in life may help mitigate cognitive decline and frailty in aging populations [152].

In summary, the MGBA throughout the life cycle underscores the importance of early life microbiome in shaping brain health and cognitive resilience in aging. Focusing on gut health during critical developmental windows could be key to reducing the risk of neurodegenerative diseases later in life.

## How Does Gut Microbiome Dysbiosis Affect the Gut-Brain Axis?

Gut microbiome dysbiosis has been increasingly associated with several neurodevelopmental and neurodegenerative disorders. Disruptions in the gut microbiome can affect brain function through mechanisms such as neuroinflammation, altered neurotransmitter synthesis, and immune system modulation. [28, 96, 177–179]. This section explores how gut dysbiosis leads to abnormal brain function in various disorders, focusing on depression, anxiety, neurodevelopmental disorders, and neurodegenerative diseases. Table 1 summarizes the impact of gut microbiome dysbiosis on different neurological disorders. 

**Table 1:** Summary of key findings and mechanisms linking gut microbiome dysbiosis to neurological disorders

Disorders	Key Findings	References
Autism spectrum disorder (ASD)	- Gut dysbiosis linked to increased *Clostridia* and decreased beneficial *Bifidobacterium* - This imbalance led to neuroinflammation, disrupted serotonin and GABA signaling - Early life microbiome imbalances may have a long-lasting effect on brain development and shape neural circuits involved in behavior, emotion, and cognition - Reduction in bile-metabolizing *Bifidobacterium* and *Blautia* species which is linked to deficiencies in bile acid and tryptophan metabolism, leading to gastrointestinal dysfunction and impaired social interactions in BTBR mice	[151, 180, 181]
Attention-deficit hyperactivity disorder (ADHD)	- Dysbiosis affects neurotransmitter production (serotonin, dopamine) - Reduced SCFAs and neuroinflammation contribute to cognitive deficits - Impaired gut-brain signaling influences attention and behavior	[182, 183]
Parkinson’s disease (PD)	- Early gut pathology, including α-synuclein in the enteric nervous system (ENS) - Dysbiosis with increased *Enterobacteriaceae* and decreased *Prevotellaceae* linked to PD severity - Microbiome shifts may serve as early diagnostic markers	[184–186]
Alzheimer’s disease (AD)	- Gut-derived metabolites trigger the NLRP3 inflammasome, leading to amyloid-β accumulation and tau/Aβ42 AD hyperphosphorylation - Dysbiosis associated with amyloid plaque overproduction and neuroinflammation	[187–191]
Multiple sclerosis (MS)	- Dysbiosis with *Akkermansia muciniphila* and *Acinetobacter calcoaceticus* leads to impaired Treg function and increased pro-inflammatory T cell responses - Higher levels of *Fusobacteria* may be linked to increased risk of MS relapses - FMT from MS patients exacerbates disease in mice	[192, 193]
Depression and anxiety	- Dysbiosis leads to serotonin depletion via impaired tryptophan metabolism - Neuroinflammation and gut-derived LPS can cross the BBB and activate microglia, leading to release of pro-inflammatory cytokines - Gut dysbiosis reduces GABA production and increases glutamate activity, heightening anxiety - HPA-axis dysregulation increases cortisol levels, perpetuating stress and inflammation	[28, 32, 33, 45, 48, 67, 151, 176, 194]
Brain tumors	- Microbiome influences tumor progression and immune responses Notable variations in the percentage of *Firmicutes* to *Bacteroides*, beta diversity, and the proportional prevalence of *Verrcomicrobia* and *Akkermansia* in stool samples from glioma patients and mice models compared to healthy controls	[195, 196]

### Neurodevelopmental Disorders

#### Autism Spectrum Disorder (ASD)

Gut dysbiosis in individuals with ASD has been consistently linked to significant changes in microbial composition, including a higher abundance of *Clostridia* species and a reduction in beneficial bacteria such as *Bifidobacterium* [180]. These microbial imbalances are thought to contribute to neuroinflammation, a key feature in ASD pathophysiology. Dysbiosis alters the gut-brain communication pathways, leading to disruptions in the synthesis of critical neurotransmitters such as serotonin and GABA, both of which are crucial for mood regulation, social behavior, and cognitive function. Serotonin, for instance, regulates brain circuits involved in social communication, and GABA helps maintain the balance between excitatory and inhibitory signaling in the brain [180]. In line with these findings, Golubeva et al. reported that a reduction in bile-metabolizing *Bifidobacterium* and *Blautia* species is associated with impaired bile acid and tryptophan metabolism, leading to gastrointestinal dysfunction and deficits in social interactions in mice model [181].

Recent research indicates that early life microbiome imbalances may have a long-lasting effect on brain development and shape neural circuits involved in behavior, emotion, and cognition. This altered brain circuitry could increase susceptibility to ASD-related symptoms, such as difficulties in social interaction and repetitive behaviors. Recent study [151] emphasizes the critical role of early life microbiome in shaping brain circuits involved in behavior and stress regulation.

#### Attention-Deficit Hyperactivity Disorder (ADHD)

Gut microbiome can also affect ADHD. Dysbiosis reduces the production of serotonin and dopamine, both important for regulating attention, mood, and behavior. Low levels of these neurotransmitters can contribute to ADHD symptoms like hyperactivity and inattention [182].

Dysbiosis also reduces SCFAs, such as butyrate, which are needed for brain health. This leads to issues with attention and cognitive function. Moreover, inflammation caused by dysbiosis can worsen ADHD symptoms. Inflammatory molecules like IL-6 and TNF-α disrupt brain areas responsible for focus and impulse control [183].

### Neurodegenerative Disorders

#### Parkinson’s Disease (PD)

PD may start in the gut, with α-synuclein pathology originating in the ENS [184]. Early symptoms, such as constipation, can appear years before the characteristic motor symptoms of PD [185]. Gut microbiome imbalances, particularly an increase in *Enterobacteriaceae* and a decrease in *Prevotellaceae*, have been linked to the severity of PD symptoms. These microbial shifts could serve as early indicators for PD, with recent studies showing that the density of *Prevotellaceae* and constipation status could diagnose PD with 90.3% specificity [186]. This highlights the potential of microbiome profiling as an early diagnostic tool for PD.

#### Alzheimer’s Disease (AD)

The pathogenesis of AD is also believed to be closely related to the microbiome; metabolic compounds derived from the microbiome have been linked to triggering the NLRP3 inflammasome pathway, amyloid-β accumulation, and phosphorylated tau/Aβ42 AD markers [187, 188]. The accumulation of Aβ deposits subsequently triggers the release of several proinflammatory molecules, leading to neuroinflammation in the progression of AD [188]. Additionally, it has been documented that *H. pylori* causes the hyperphosphorylation of tau protein and the release of amyloids and inflammatory molecules [189, 190]. This suggesting that modulating the gut microbiome may offer a therapeutic strategy for combating neurodegeneration in AD [191].

#### Multiple Sclerosis (MS)

In MS, the gut microbiome plays a key role in immune regulation. Studies have found that *Akkermansia muciniphila* and *Acinetobacter calcoaceticus* are more common in the gut microbiome of MS patients. These bacteria can impair the function of Treg cells and increase the activity of pro-inflammatory T cells in laboratory experiments [192]. Other research has linked higher levels of *Fusobacteria* to an increased risk of MS relapses, suggesting that changes in the microbiome could make the disease worse [193]. Animal studies have shown that fecal microbiota transplantation (FMT) from MS patients worsens symptoms in mice. Mice receiving FMT from MS patients developed more severe experimental autoimmune encephalomyelitis (EAE), a model for MS, and had fewer anti-inflammatory Treg cells compared to mice receiving FMT from healthy donors. This highlights how the gut microbiome influences immune function and may contribute to MS progression [192].

### Mental Health Disorders: Depression and Anxiety

Depression and anxiety are among the most thoroughly investigated mental health conditions linked to MGBA. Emerging evidence highlights the profound influence of gut microbiome on neurochemical balance, inflammation, and stress regulation, which are all central to these disorders. One major mechanism involves the depletion of beneficial bacteria such as *Bifidobacterium* and *Lactobacillus*, which impairs tryptophan metabolism. Tryptophan is essential for synthesizing serotonin, a neurotransmitter crucial for mood regulation. Dysbiosis not only reduces serotonin production but also activates the kynurenine pathway, producing neurotoxic metabolites linked to depressive symptoms [45, 151]. Additionally, the reduction of SCFAs like butyrate compromises BBB integrity and promotes neuroinflammation, further exacerbating mood disorders [32, 48]. Additionally, neuroinflammation plays a critical role in both depression and anxiety. Increased levels of LPS from Gram-negative bacteria can cross the BBB and activate microglia leading to the release of pro-inflammatory cytokines such as IL-6 and TNF-α. This inflammatory response contributes to the neurobiological basis of these disorders. Dysbiosis also disrupts the synthesis of GABA, an inhibitory neurotransmitter that helps counterbalance excitatory signals. Reduced GABA and elevated glutamate activity heighten anxiety and stress responses [28, 33]. Moreover, gut dysbiosis affects the HPA-axis, increasing cortisol production and perpetuating a cycle of chronic stress and inflammation [67, 194]. Similarly, disruptions in myelination processes, particularly in the PFC, further link dysbiosis to cognitive and emotional deficits observed in mood disorders. The PFC plays a central role in higher cognitive functions such as decision-making, emotional regulation, and stress response. In dysbiotic conditions, gut-derived metabolites like SCFAs, which are crucial for maintaining myelination, are reduced. This deficiency leads to impaired oligodendrocyte function and delayed myelination of the PFC, which directly impacts brain circuits responsible for emotional processing and behavioral responses to stress [176]. As a result, the altered myelination observed in dysbiosis contributes to increased vulnerability to anxiety and depression by disrupting the brain’s capacity to regulate emotional responses and cognitive functions.

### Brain Tumor

The gut microbiome may influence glioma progression by altering immune responses and metabolic pathways [195]. Notable variations were found in the percentage of *Firmicutes* to *Bacteroides*, beta diversity, and the proportional prevalence of *Verrcomicrobia* and *Akkermansia* in stool samples from glioma patients and mice models compared to healthy controls [196].

All things considered, gut-modulated physiological and cellular alterations are probably exceedingly intricate and multidimensional, even if the role of the microbiome in CNS disorders is quite relevant for redesigning treatment design. Subsequent research efforts should examine the applicability of correlated observations and confirm the consistency of current experimental results. Preclinical and clinical studies for CNS disorders frequently include distracting factors like antibiotics and medications, which when combined with other variables like dietary variations and geographic location, will have a substantial impact on gut composition outside of the therapy or study model. This is another important point to keep in mind. Further investigations are necessary to determine the relationship between the gut microbiome and neurodegenerative or neurodevelopmental illnesses, as well as how CNS disorders may affect the gut microbiome's makeup. Furthermore, there is a compelling opportunity to use our expanding understanding of gut microbiome as indicative biomarkers for illnesses; nonetheless, more varied patient cohorts need to be investigated in order to confirm that microbial biomarkers are applicable to various racial and geographic backgrounds.

## How to Manage?

### Antibiotics

It has been demonstrated that taking antibiotics can change the makeup of gut bacteria, which can have a beneficial or bad impact on brain functions. Several studies confirmed that a negative impact was shown with increased serum levels and associated with cognitive and anxiety-like behaviors [28]. The kind and purposes of antibiotics, however, determine these unfavorable side effects. Amoxicillin and clarithromycin, for instance, decreased *H. pylori* load, which has a beneficial effect on brain functions in AD patients [191, 197]. In the PD rat model, the administration of non-absorbable antibiotics minimizes the death of dopaminergic neurons caused by 6-hydroxydopamine (6-OHDA), the generation of proinflammatory mediators in the striatum, and the extent of motor impairment [198]. Additionally, the injection of rapamycin, minocycline [199, 200], and rifampicin [198, 201] decreased the microglial activity, inflammatory cytokines, and Aβ level in the AD animal model [202].

### Probiotic

Probiotics are referred to as “live microorganisms that, when consumed in sufficient amounts, provide beneficial effects on the host.” Probiotics are frequently found in foods or dietary supplements that we regularly eat, such yogurt. A growing number of “designer probiotics”—which are genetically modified to optimize the advantageous properties of particular bacteria—have been released; these supplements are frequently found in more conventional pharmaceutical forms [203]. A number of studies have notably shown the positive impacts of probiotic administration of *Bifidobacteria* and *Lactobacillus*, which raises GABA levels and the expression of neurotropic factors. These effects include a decrease in the frequency of attacks of seizures in patients with drug-resistant epilepsy [204], improvement of patients’ difficulties in learning and spatial memory through the use of a fermented milk mixture [205], and a decrease in motor impairment and dopaminergic neurodegeneration in MitoPark PD mouse models [206]. Moreover, a novel probiotic formulation called SLAB51 was able to restore phenotypic abnormalities in motor activity when administered to a mouse PD-model and boost cell survival in a human in vitro PD model by lowering oxidative stress and neuronal death [207]. Of all the probiotics under investigation, the majority of research focuses on how probiotic *Lactobacillus Plantarum* affects a number of CNS conditions, such as major depressive disorder [208, 209], stress [210, 211], ADHD [212], and ASD [213, 214].

Thus far, the findings indicate significant promise for using beneficial gut microbes as new treatment approaches for neurological disorders. The content, stability, and legitimacy of probiotics vary greatly, and there is no agreement on the best way to take them, how long to take them, or which strains to employ. In addition, host colonization resistance raises serious questions about the utility of probiotic-based treatments in general. While the beneficial effects are not persistent because probiotics cannot inhabit in the acidic conditions of the gut for long periods [30], it may be argued that probiotics have a therapeutic advantage in that they do not permanently modify the cell environment but instead only need regular intake as needed.

### Prebiotic

Prebiotics are indigestible dietary fibers that improve the health of the host by promoting the development and activity of certain gut microbiomes, particularly *Lactobacillus* and *Bifidobacterium* [28, 215]. Prebiotics are mostly made up of resistant starch, fructo-oligosaccharides (FOS), and galacto-oligosaccharides (GOS), which are specifically used by gut microbes as the major source of nutrition and provide health advantages. Prebiotics, which are frequently present in fruits, vegetables, cereals, and human milk, have the benefit of influencing the gut microbiome on a broader scale. For example, a decrease in dietary fiber by the gut microbiome leads to a significant increase in immune-boosting *Faecalibacterium prausnitzii* and SCFA butyrate, both important for gut health. In contrast, a high-fat, low-fiber, animal-based diet reduces the number of beneficial bacteria and promotes the growth of bile-tolerant microbes [216–218].

Prebiotics have been shown to enhance cognitive performance and guard against neurological conditions including PD [219, 220], AD [221], ASD [222], IBS [223], and dementia [224]. Burokas et al. demonstrated that FOS and GOS improve depression- and anxiety-like behaviors in a chronic stress mouse model by targeting the MGBA [225]. Building on this, Vijaya et al. recently reported that these prebiotics can also mitigate the detrimental effects of long-term high-fat diet consumption in aging mice, including anxiety, cognitive impairment, and microglial dysfunction [226]. In mice subjected to stress, prebiotics were able to decrease proinflammatory cytokines and corticosterone produced by chronic stress as well as boost cecal SCFAs (acetate and propionate). Likewise, prebiotic Bimuno-galacto-oligosaccharides (B-GOS) therapy improved mental performance and markedly reduced triggering of microglia and the production of CD32, CD68, SOCS3, iNOS, and IL-6 in rats [227].

**Fig. 4:**
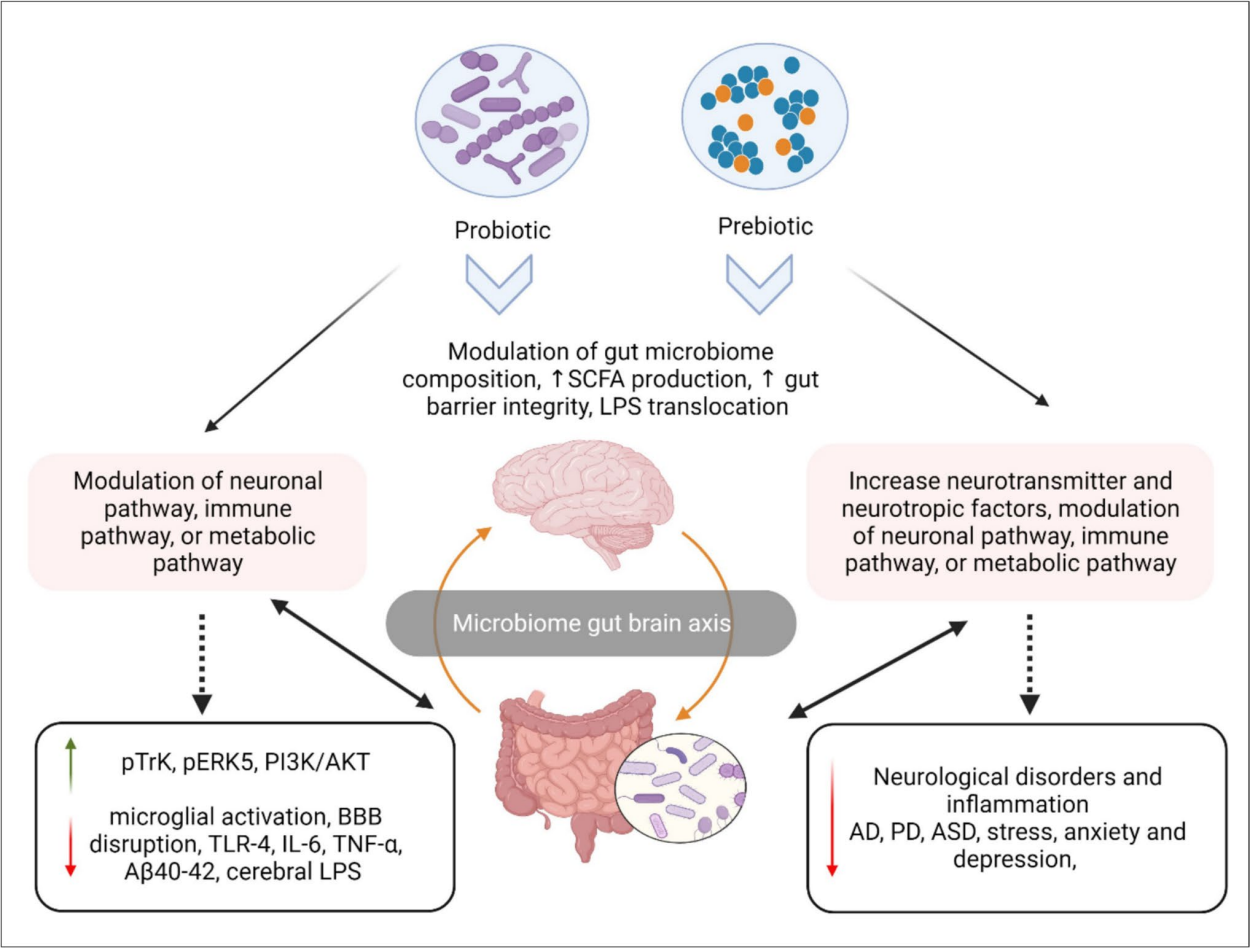
Impact of prebiotics and probiotics on microbiome gut-brain axis improvement. Probiotics, prebiotics, or a combination of the two have been shown to ameliorate neurological problems by modifying immunological, metabolic, and neuronal pathways; lowering intestinal permeability; boosting the synthesis of SCFAs and neurochemicals; and modifying the makeup of gut microbiome (modified from [ 28 ], created with BioRender)

In summary, the illustration (Figure 4) clarified how probiotics and prebiotics enhance brain function by regulating the microbiome and gut-brain axis.

### Synbiotic and Postbiotic

Synbiotics are a mixture of probiotics and prebiotics, in which the prebiotics serve as a reservoir of fermentable fiber to complement and improve the longevity of the probiotics [30, 228]. Postbiotics are fermentation byproducts of bacteria that include bioactive substances with beneficial properties like SCFAs and gut peptides. Postbiotics have a clear benefit over probiotics regarding longer shelf life and a safer profile; however, better-defined targets are still needed [229]. Interestingly, studies have discovered that higher-fiber diets produce more butyrate, which has neuroprotective properties and improves neuron plasticity, as well as a larger abundance of beneficial taxa and a more diverse and richer gut microbiome. Consequently, nutrition has a big impact on how CNS illnesses turn out. For instance, in a study involving PD mice, the experimental group experienced a markedly lower loss of dopaminergic neurons and a deterioration in motor function than the control group [230]. Furthermore, it has been discovered that high-fat, low-carb, and sufficient protein ketogenic diets can decrease the frequency of seizures in epilepsy patients by raising GABA and glutamate levels [231]. It is interesting to note that these findings apply to a variety of illnesses, including gliomas. For instance, in the gliomas animal model, treated with a glutamine antagonist, 6-diazo-5-oxo-l-norleucine, and a calorically restricted ketogenic diet concurrently showed massive death of tumor cells with no apparent toxicity [232]. According to a systematic study, probiotics, prebiotics, and synbiotics are effective treatment approaches for enhancing cognitive abilities, behavioral symptoms as well as psychological symptoms in dementia patients [224]. Many clinical investigations are presently being carried out to better examine the impact of prebiotics and synbiotics in various neurological illnesses in order to transition from lab to clinic. For instance, the impact of GOS on cortical stimulation and plasticity, anxiety regulations, and thought control capability is being examined in a significant ongoing longitudinal investigation named “The Role of the Microbiota-gut-brain Axis in Brain Development and Mental Health.” Moreover, a phase III clinical trial is currently being conducted on a different research, named “Mediterranean-DASH Diet Intervention for Neurodegenerative Delay,” to examine the impact of a hybrid diet on the cognitive decline of older, overweight people who had inadequate nutritional intake in the past [31].

There is undoubtedly growing interest in the advantages that healthy diets provide for CNS therapy interventions and consequences, despite the difficulty in precisely targeting particular gut microbes and the demand for an effective discovery of how commensal bacterial populations’ initial states may affect the efficacy of dietary mediations.

### Fecal Microbiota Transplantation (FMT)

FMT has been demonstrated to halt the course of seizures [233] and MS [234] for a considerable amount of time and to temporarily lessen PD symptoms (such as neuroinflammation and leg tremors) [235, 236]. Additionally, improved spatial learning and memory were demonstrated by the transplantation of feces from senescence-resistant mice into models of AD animals [237]. Given its capacity to transmit both immune-regulatory and disease-promoting bacteria, FMT is currently surrounded by confusion due to unclear classifications of a favorable microbiome and the need to confirm its advantages and long-term impacts. New research looked at FMT and how it affected mice with depression. When compared to the control group, mice who receive fecal microbiota from chronic unpredictable mild stress (CUMS) mice exhibited higher levels of depression and anxiety-like behaviors. Furthermore, increased amounts of TNF-α, IFN-γ, and indoleamine 2,3 dioxygenase 1 (IDO1) were detected in the recipient mice’s hippocampal regions. This suggests that the gut microbiome influences the inflammatory response in the hippocampal regions by disrupting the microbiome-gut-brain axis, which in turn intensifies symptoms of anxiety and depression [238]. Moreover, FMT-treated MS patients had improved disease progression and symptoms [234, 239]. In a study demonstrating the effect of FTM from AD patients to GF mice found that mice behavior was affected by this transplantation but some metabolites such as taurine, valine, and γ-aminobutyrate much less prevalent in the stool of mice that received a microbiota transplant from the diseased patient [240]. In a reported case report, the gastrointestinal problems and tremors in the legs of the 71-year-old PD patient improved after receiving FMT from young, healthy donors [235].

Finally, FMT’s lack of specificity outweighs its great potential to positively alter the gut microbiome composition of unhealthy individuals. It is unclear at this point whether FMT will be enough as a stand-alone treatment; this answer will probably rely on the nature of the disease and the microbes that affect it.

## Therapeutic Approaches and Challenges

While probiotics, prebiotics, and FMT show promise in modulating the MGBA and improving mental health outcomes, several significant challenges limit their clinical application and effectiveness.

### Variability in Microbiome Composition

The gut microbiome is highly individualized, shaped by genetics, diet, environment, and lifestyle. This variability complicates the development of standardized treatments, as the same probiotic or prebiotic treatment may not yield consistent results across different individuals. For instance, a probiotic strain that proves effective in one person may have no impact on another due to differences in their microbiome composition. Personalized approaches, considering the individual’s unique microbiome, are critical for enhancing treatment outcomes. Recent studies [194] emphasize that the baseline microbiome composition significantly affects the efficacy of interventions, underscoring the need for tailored treatments.

### Standardization and Efficacy

Current probiotic and prebiotic formulations vary widely in strain composition, dosage, and viability. This lack of standardization leads to inconsistent clinical results, making it difficult to determine the optimal dose, duration, and specific strains needed to effectively modulate the MGBA. To improve reliability and clinical applicability, rigorous clinical trials must be conducted to establish clear guidelines for formulation, dosage, and treatment protocols. Furthermore, while some studies show promise for probiotics in conditions like depression and anxiety, results have been inconsistent, highlighting the need for more controlled, large-scale trials.

### Ethical and Practical Considerations in FMT

There are several ethical and safety concerns remaining upon using FTM. Ethical issues related to donor selection and the risk of pathogen transmission have yet to be fully addressed [241, 242]. The long-term effects of FMT on the recipient’s microbiome and overall health are still unclear, making it crucial to develop standardized safety protocols for donor screening and post-treatment monitoring [236]. Furthermore, the lack of consensus on optimal treatment protocols, including the preparation, storage, and administration of fecal transplants, complicates its widespread clinical use. While FMT offers significant promise in restoring microbial balance, its effectiveness remains highly dependent on both the recipient’s microbiota composition and the specific microbial strains used in the transplant [236].

## Research Gaps and Future Directions

Although the MGBA is increasingly recognized as a potential therapeutic target, more research is needed to address the gaps in our understanding of its role in mental health. Much of the existing literature relies on animal models, which may not accurately reflect human physiology. Additionally, the long-term effects of dietary interventions, prebiotics, and probiotics on gut microbiome stability and overall health are still under investigation. To better harness the therapeutic potential of microbiome-based treatments, personalized medicine approaches should be prioritized, integrating microbiome profiling and other biomarkers to tailor interventions to individual patients.

## Conclusion

The gut and brain are connected in a complex bidirectional pathway called gut-brain axis. Growing findings on the impact of intestinal inflammation on the neurological system are crucial for connecting the missed dots between the two regions and further understanding the MGBA’s synergistic connection. This review discusses the reported connections and pathways that are involved in MGBA focusing on the important role of gut microbiome and their metabolites. Subsequently, it centralizes the attention toward the critical roles of the gut microbiome in brain development in early life and throughout the course of life and how microbiome dysbiosis affects MGBA and influences neurological diseases. Finally, sheds light on how to manage this disease’s conditions through the rebalance of the gut microbiome.

## Data Availability

No datasets were generated or analysed during the current study.
